# Allergic Disorders and Risk of Anemia in Japanese Children: Findings from the Japan Environment and Children’s Study

**DOI:** 10.3390/nu14204335

**Published:** 2022-10-17

**Authors:** Limin Yang, Miori Sato, Mayako Saito-Abe, Yumiko Miyaji, Mami Shimada, Chikako Sato, Minaho Nishizato, Natsuhiko Kumasaka, Hidetoshi Mezawa, Kiwako Yamamoto-Hanada, Yukihiro Ohya, on behalf of the Japan Environment and Children’s Study (JECS) Group

**Affiliations:** Medical Support Center for the Japan Environment and Children’s Study, National Center for Child Health and Development, Tokyo 157-8535, Japan

**Keywords:** children, birth cohort, allergy, anemia, health

## Abstract

Previous epidemiological studies have reported an increased risk of anemia in people with allergic disorders. However, previous studies have followed a cross-sectional design. The aim of this study was to investigate the association between the two conditions with a cohort dataset. We used data of 80,943 children in the Japan Environment and Children’s Study, the largest birth cohort in Japan. The association between anemia and allergic disorders was evaluated with a logistic regression model and propensity score analysis. After adjusting for potential confounders, children with asthma (odds ratio [OR], 1.85; 95% confidence interval [CI], 1.32–2.60), atopic dermatitis (OR, 2.18; 95% CI, 1.66–2.85), allergic rhinitis (OR, 1.35; 95% CI, 1.05–1.74), allergic rhinoconjunctivitis (OR, 2.95; 95% CI, 1.91–4.54), and food allergies (OR, 1.92; 95% CI, 1.44–2.56) at 2 years of age predicted high odds of developing anemia in the next year. Any allergy at 2 years of age was associated with an increased risk of anemia at the age of 3 years (OR, 1.80; 95% CI, 1.41–2.29). The findings remained stable in the propensity score analysis. Results suggest that allergic diseases were related to caregiver-reported anemia in children.

## 1. Introduction

Anemia is a condition characterized by a decreased hemoglobin level, as well as insufficiency in red blood cell mass, resulting in the inability to carry oxygen to peripheral tissues [1,2]. Iron deficiency anemia and anemia of inflammation are the two major subtypes. Anemia is common in children and may cause impaired cognitive and motor development [3,4]. The common risk factors for childhood anemia include prematurity, malnutrition, drinking cow’s milk, chronic blood loss, and inflammation [5,6]. Estimates suggest that 273 million children across the world suffered from anemia in 2011 [4]. Its prevalence among Japanese children aged <5 years was estimated to be 17% in 2019, according to the World Health Organization [6].

Allergies are the most common conditions in children, with increasing prevalence rates in both children and adults in recent decades [7,8,9]. Asthma, atopic dermatitis (AD), and allergic rhinitis are hypersensitivity disorders in which chronic inflammation occurs in the lung, skin, and nasal mucosa. Chronic inflammatory conditions can cause anemia or iron deficiency by impairing the immune-mediated regulation of hepcidin and cytokines, which are important in iron homeostasis [10]. Furthermore, food avoidance in people with a food allergy may also correlate with malnutrition [11]. Hence, this implies a potential relationship between the two conditions. Previous epidemiological studies have reported an increased risk of developing anemia in people with allergies. However, existing studies could not provide a conclusion on the temporal association between allergic diseases and anemia due to the limitation of a cross-sectional design.

The ongoing birth cohort study, including around 100,000 participants in Japan, called “the Japan Environment and Children’s Study (JECS),” provided us an opportunity to explore whether allergic diseases place children at risk for the development of anemia. We designed a cohort study to examine the effect of allergic diseases, including asthma, AD, allergic rhinitis, allergic rhinoconjunctivitis, and food allergies, on the development of anemia in children.

## 2. Materials and Methods

### 2.1. JECS and Study Design

We used data from the JECS, which was a government-funded birth cohort study to evaluate the association of antenatal and postnatal environmental exposure with children growth/development and various health issues [12,13,14]. The JECS recruited 97,413 mothers (103,060 pregnancies) from 15 regional centers (Hokkaido, Miyagi, Fukushima, Chiba, Kanagawa, Koshin, Toyama, Aichi, Kyoto, Osaka, Hyogo, Tottori, Kochi, Fukuoka, and South Kyushu/Okinawa) from January 2011 to March 2014. The offspring of the pregnant women will be followed up until the children reach 13 years of age [12,13,14]. The demographic characteristics of the mothers and children of the JECS were similar to those published by Japan’s 2013 Vital Statistics Survey [13]. These details have been published elsewhere [13,14].

The JECS performed questionnaire surveys and medical examinations to obtain data on pregnant women and their children. The questionnaire surveys were performed during the first and second/third trimesters to collect data on socioeconomic status and health conditions during pregnancy [12]. After birth, multiple surveys were conducted to collect data on children’s health and development [12].

**Figure 1:**
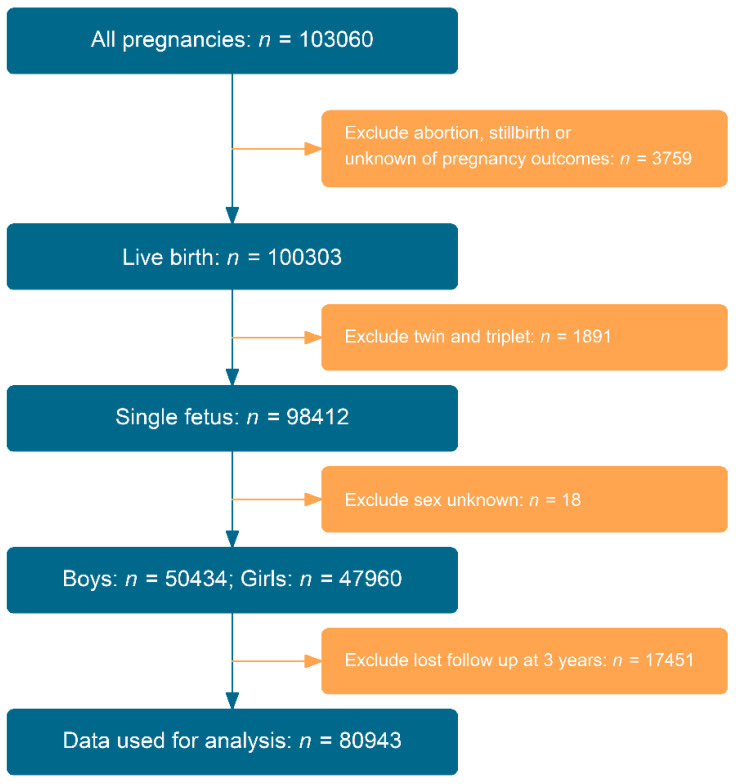
Flow chart of study cohort.

We designed this cohort study to evaluate whether children with allergic diseases at 2 years of age had increased risk of developing anemia at the age of 3 years. We excluded records with miscarriages, stillbirths, and multiple births (twins and triplets). Finally, we excluded those who were lost to follow-up at 3 years of age. Figure 1 depicts a chart showing the detailed selection of data used for analysis in this study. Data on allergic diseases and anemia were obtained from a postal questionnaire survey.

We used the dataset named jecs-ta-20190930, which was first released in October 2019 (last accessed in May 2022).

All procedures involving human subjects for the JECS protocol were reviewed and approved by the Institutional Review Board on Epidemiological Studies of the Ministry of the Environment (Ethical Number: No.100910001) and the Ethics Committees of all participating institutions [13,14,15]. Written informed consent was obtained from all participants.

### 2.2. Allergic Diseases at the Age of 2 Years

Allergic diseases were defined according to the International Study of Asthma and Allergies in Childhood (ISAAC), which was a partially modified version of the ISAAC questionnaire for 6- and7-year-old children with translation validation in Japanese [16,17,18], or a caregiver-reported and doctor-diagnosed condition. Details are described in Table S1. “Any allergy,” a composite variable, was generated, defined as the presence of any asthma, AD, allergic rhinitis, or food allergy.

### 2.3. Anemia at the Age of 3 Years and Confounding Variables

“Anemia” was defined by a positive response to the question “Has your child ever been diagnosed with anemia by a doctor since the age of 2 years?” The variables used in the analysis included dichotomous variables (smoking exposure (yes or no), maternal allergic history (yes or no), parental education levels (low vs. normal or high), family income (<4 or ≥4 million JPY per year), pet keeping (yes or no), birth weight (<2500 or ≥2500 g), sex (boys or girls), premature birth (<37 or ≥37 weeks), breastfeeding (yes or no), sleeping time (<11.5 or ≥11.5 h), kindergarten (yes or no), and parity (nulliparous or multiparous)) and a continuous variable (z score of body mass index (BMI) at 2 years). We calculated z scores for BMI using the LMS method, with age- and sex-specific values of L, M, and S obtained from the Japanese growth curve criteria [19,20,21,22].

### 2.4. Statistical Methods

Logistic regression models with anemia as a dependent variable were fitted to estimate odds ratios (ORs). Multicollinearity was checked using variance inflation factor values. We found no evidence of multicollinearity among the abovementioned covariates. Under the assumption of missing at random, multiple imputation (MI) was performed to manage missing data on allergy or other confounders. A total of 10 complete datasets were generated by MI. The model was constructed for each generated dataset and then combined to obtain a pooled OR.

The main models adjusted smoking exposure, parental education levels, family income, birth weight, sex, premature birth, breastfeeding, sleeping time, and kindergarten. After fitting the main model, we further adjusted the z score of BMI at 2 years of age to evaluate whether this variable mediates the association between allergy and anemia. We also investigated the effect of the number of comorbid allergic disorders on anemia. The covariates in the models were selected according to previous studies in which the variables were found to be associated with anemia.

We also performed a propensity score (PS) analysis. First, the PS was calculated by fitting a logistic regression model with independent variables (smoking exposure, maternal allergic history, parental education levels, family income, pet keeping, birth weight, sex, premature birth, breastfeeding, sleeping time, kindergarten, and parity). Then, a weighted logistic model was fitted using the inverse probability of treatment weighting (IPTW) approach. Furthermore, we fitted logistic models adjusting for the calculated PS as a covariate. In this process, the restricted cubic spline (with 3-knot) approach was used for the PS values to relax linear assumption in the model.

Finally, we conducted sensitivity analyses and subgroup analyses. The same models were fitted using the complete dataset. We also evaluated the effect modification by sex, birth weight, preterm birth, and feeding methods for the associations by adding the interaction term into the models. The ISAAC collected information on the severity of allergic diseases, such as sleep disturbance due to AD or having interference in daily life due to allergic rhinitis. Therefore, we conducted the subgroup analysis on those with AD or allergic rhinitis to identify the effect of the severity of diseases on anemia. Another subgroup was a cohort that excluded children diagnosed with anemia by a doctor at 1 year of age, according to the caregiver’s report.

All results from the logistic models are presented with OR with 95% confidence intervals (CIs). Description analysis and model fitting were performed using the R version 4.3.0 software (Institute for Statistics and Mathematics, Vienna, Austria; www.r-project.org, accessed on 10 December 2020).

## 3. Results

### 3.1. Baseline Characteristics

After data selection, we collected 80,943 records for this analysis. Table S2 summarizes the baseline characteristics of these participants. The study cohort consisted of 41,483 (51.2%) boys and 39,460 (48.8%) girls. The prevalence rates of asthma, AD, allergic rhinitis, allergic rhinoconjunctivitis, and food allergy were 7.5, 13.0, 28.2, 2.9, and 11.1%, respectively. Overall, 34,967 (43.7%) children suffered from an allergy in the study cohort. Anemia at the age of 3 years was reported in 282 (0.3%) children.

### 3.2. Relationship between Allergic Disorders and Anemia

The association between allergic diseases at 2 years of age and the development of anemia at 3 years of age is presented in Table 1. In the main model, after adjusting for potential confounders, children with asthma (OR, 1.85; 95% CI, 1.32–2.60), AD (OR, 2.18; 95% CI, 1.66–2.85), allergic rhinitis (OR, 1.35; 95% CI, 1.05–1.74), allergic rhinoconjunctivitis (OR, 2.95; 95% CI, 1.91–4.54), and food allergy (OR, 1.92; 95% CI, 1.44–2.56) at 2 years of age predicted a high odds of developing anemia in the next year. Children with any allergy at the age of 2 years were 1.80 (95% CI, 1.41–2.29) times more likely to develop anemia at the age of 3 years than normal children. Further adjusted z scores of BMI at 2 years of age did not significantly change the abovementioned OR.

The risk of anemia development at the age of 3 years increased with an increase in the number of comorbid allergic diseases. The odds of developing anemia was 1.38 (95% CI, 1.05–1.82) times higher in the presence of a single allergic disease than that with no allergy. However, those with comorbid allergic diseases were 2.84 (95% CI, 2.12–3.84) times more likely to develop anemia than those without allergies.

### 3.3. Propensity Score Analysis

In the IPTW approach, we first checked the balance between the two exposure groups after weighting with standardized mean differences (SMDs) (Table S3). All values of SMD were <0.1, suggesting that after weighting, the distribution reached a balance between the two groups for each atopic disease. ORs from the weighted logistic regression are presented in Table 2. Any allergy was associated with increased odds of anemia at 3 years of age (OR, 1.87, 95% CI, 1.42–2.45). Children with asthma (OR, 1.62; 95% CI, 1.09–2.41), AD (OR, 2.36; 95% CI, 1.76–3.16), allergic rhinitis (OR, 1.35; 95% CI, 1.02–1.78), allergic rhinoconjunctivitis (OR, 2.99; 95% CI, 1.80–4.97), and food allergies (OR, 1.95; 95% CI, 1.42–2.68) had significantly higher risk of developing anemia than children without these conditions.

When PSs were adjusted as a continuous variable in the models, the ORs remained stable.

### 3.4. Sensitivity Analysis and Subgroup Analysis

As listed in Table S4, the estimates from the models using the complete dataset were very similar to those presented in Table 1.

There was no effect modification by sex, birth weight, preterm birth, and feeding methods. All the interaction terms in the models were nonsignificant.

The ORs from the subgroup analysis are presented in Table S5 and Table S6. Children with AD with sleep disturbance had increased risk of developing anemia compared with those without sleep disturbance (OR, 1.95; 95% CI, 1.22–3.10). Similarly, children with severe interference in daily life due to rhinitis were more likely to suffer from anemia than children with mild rhinitis (OR, 2.52; 95% CI, 1.26–5.02) (Table S5). When we excluded children having been diagnosed with anemia by a doctor at the age of 1 year, the ORs of allergic diseases on anemia remained significant (Table S6).

## 4. Discussion

Using the largest birth cohort in Japan, this cohort analysis found that anemia in children aged 3 years was more common in those with caregiver-reported allergic conditions at the age of 2 years than in those without allergic diseases. Moreover, we observed that the greater the number of comorbid allergic diseases, the higher the risk of developing anemia. To our knowledge, our study provides the largest cohort analysis addressing the relationship between the two conditions in the pediatric population.

Publications regarding the association of allergy with anemia are rare. A cross-sectional study conducted by Drury et al. using data from two large US surveys reported that children with AD, asthma, hay fever, and food allergy were more likely to develop anemia [23]. Another cross-sectional study on an Asian pediatric population conducted by Rhew and Oh analyzed data from the Health Insurance Review and Assessment Service of South Korea, and reported similar association between atopic diseases and anemia [24]. The same study group further documented this association in both young individuals and adults using a PS analysis [25]. Furthermore, Chang et al. compared the prevalence of anemia in children with both controlled and uncontrolled asthma (severe asthma) and found that anemia was more common in patients with uncontrolled asthma [10]. The results of the present study are concordant with these published data. Moreover, our cohort study progresses further than previous research, as it provides insight into the directionality of the relationship between allergy and anemia.

The reasons for the Increased risk of anemia in children with allergic diseases are not completely understood. Children with allergic diseases are at risk of developing chronic inflammation, which can cause anemia of chronic disease, the most frequent anemic entity observed in chronically ill patients [23,26,27]. It has been observed that inflammatory mediators inhibited erythroid cell differentiation, shortened erythrocyte half-life, and suppressed erythropoietin response to anemia, resulting in anemia of inflammation [28]. For instance, interleukin-6 (IL-6), IL-1, and activin B were found to stimulate hepcidin production, which is important in iron homeostasis [28]. Corticosteroids further contribute to anemia by increasing the odds of gastrointestinal bleeding [29]. Furthermore, avoidance of suspected food products by children with a food allergy can cause multiple nutrient deficiencies. Studies exploring nutritional risks in children with food allergies indicated that those with wheat and soy allergies were more likely to suffer inadequate intake of iron, zinc, and vitamin B6 [30,31]. Further studies by collecting data on nutrients, laboratory markers of nutritional status, and comorbidities are required to validate the above-described findings.

Our study highlights the importance of the management of anemia in children with allergy, considering that anemia may cause problems in the growth and development of children. Counseling on dietary habits and individualized nutritional advice will be beneficial for children with a food allergy [11]. In addition to nutritional management of children with a food allergy, increased clinician awareness of screening and monitoring of anemia in children with allergy may relieve the disease burden of both conditions.

A strength of this study was the large sample size, which allowed robust estimation of ORs and adjusting for more covariates in the models. Moreover, as described earlier, superior to a cross-sectional study, a cohort design allows us to clearly demonstrate the temporal association between the two conditions. Although we did not exclude the findings of anemia at the age of 2 years, due lack of data in JECS dataset, it is unlikely to explain the increased anemia risk in children with allergic diseases with reverse causation (anemia at the age of 1 year causes allergic diseases thereafter) given the pathophysiology of anemia and allergic diseases. Furthermore, although this is an observational study, we used the PS approach to balance the confounding between allergy and non-allergy groups. The PS approach is widely used in observational studies. A previous study indicated that for data with a small number of events, the PS analysis generates a more robust and precise effect estimation than conventional regression models [32].

Despite these advantages, there were some limitations in this study. First, although the dataset was obtained from the largest birth cohort in Japan, generalization to other populations must be done with caution, because, strictly speaking, the JECS is not a population-based investigation. Moreover, there still exists a selection bias in this study. When the data used for this analysis were compared with those deleted due to being lost to follow-up at 3 years of age, mild differences could be observed in some variables (Table S7 and Table S8). For instance, compared with patients lost to follow-up, participants in the study cohort were more likely to be well-educated and have higher family incomes. Although the ORs may be influenced by these differences, the number of excluded data was not large. Hence, excluding these data will not cause tremendous changes in results. The second limitation is the lack of physician diagnosis for allergic diseases. We defined allergic diseases based on the caregiver’s report from self-administered questionnaires; thus, the study may be subject to recall bias and misclassification, which may dilute the observed associations. However, the ISAAC is validated and widely used across the world, and studies have found good agreement between caregiver-reported and doctor-diagnosed allergic diseases. Third, we did not define anemia according to laboratory values of blood tests, as blood tests were not performed in children aged 3 years. Although misclassification in anemia may introduce bias into the study, errors in outcome classification have little impact on the estimation of association and bias toward the null. Therefore, we believe that the statistical significance of the associations would not disappear even if blood tests were used for diagnosis. Another limitation is that the JECS data did not query about anemia by subtypes, which prevented us from distinguishing between iron deficiency anemia and anemia of inflammation. Finally, although we adjusted for several confounders in the models, other factors could not be adjusted in this study due to lack of data in the JECS, such as nutrients and family history of anemia.

## 5. Conclusions

In conclusion, our findings indicated that allergic diseases were related to caregiver-reported anemia in children. Considering the high risk of negative effects on growth/development and quality of life in children with anemia, increased clinician awareness of screening and monitoring of anemia may relieve the disease burden in children with allergic diseases. In addition, nutritional management for food allergy should be carefully planned, avoiding unnecessary dietary restrictions. Further studies are required to elucidate the pathophysiological mechanisms underlying the association between the two conditions.

## Figures and Tables

**Table 1:** Association between atopic diseases at 2 years of age and anemia at 3 years of age in Japanese children.

		Model 1 ^#^			Model 2 ^$^		
			95% CI			95% CI	
Outcome Events		ORs	Lower	Upper	ORs	Lower	Upper
Asthma	Yes vs. No	1.85	1.32	2.60	1.91	1.36	2.69
Atopic dermatitis	Yes vs. No	2.18	1.66	2.85	2.17	1.65	2.84
Rhinitis	Yes vs. No	1.35	1.05	1.74	1.36	1.06	1.75
Allergic rhinoconjunctivitis	Yes vs. No	2.95	1.91	4.54	2.94	1.91	4.53
Food allergy	Yes vs. No	1.92	1.44	2.56	1.90	1.42	2.53
Any allergy	Yes vs. No	1.80	1.41	2.29	1.81	1.42	2.30
Atopic diseases, No.	1 vs. 0	1.38	1.05	1.82	1.39	1.05	1.83
	>1 vs. 0	2.84	2.12	3.82	2.86	2.13	3.84

Abbreviations: OR, odds ratio; CI: confidence interval; BMI, body mass index. ^#^ The model adjusted smoking exposure, parental education levels, family income, birth weight, sex, premature birth, breastfeeding, sleeping time, and kindergarten. ^$^ Except those adjusted in model 1, the models further adjusted the z scores of BMI at 2 years.

**Table 2:** Association between atopic diseases at 2 years of age and anemia at 3 years of age in Japanese children assessed using PS analysis.

			95% CI			95% CI	
Outcome Events		OR ^ab^	Lower	Upper	OR ^ac^	Lower	Upper
Asthma	Yes vs. No	1.62	1.09	2.41	1.75	1.11	2.76
Atopic dermatitis	Yes vs. No	2.36	1.76	3.16	2.35	1.75	3.17
Allergic rhinitis	Yes vs. No	1.35	1.02	1.78	1.39	1.05	1.83
Allergic rhinoconjunctivitis	Yes vs. No	2.99	1.80	4.97	2.88	1.77	4.69
Food allergy	Yes vs. No	1.95	1.42	2.68	2.04	1.47	2.82
Any allergy	Yes vs. No	1.87	1.42	2.45	1.86	1.41	2.43

Abbreviations: OR, odds ratio; CI, confidence interval; PS, propensity score. IPTW, inverse probability of treatment weighting. ^a^ The PSs were calculated by fitting a logistic model with independent variables (smoking exposure, maternal allergic history, parental education levels, family income, pet keeping, birth weight, sex, premature birth, breastfeeding, sleeping time, kindergarten, and parity). ^b^ A logistic model with IPTW. ^c^ PSs were adjusted as a covariate in the models.

## Data Availability

Details are presented in supplements.

## Supplementary Materials

The following supporting information can be downloaded at: https://www.mdpi.com/article/10.3390/nu14204335/s1, Table S1: Definitions of allergic diseases, Table S2: Baseline characteristics of the study cohort; Table S3: Standardized mean difference after weighing in the IPTW process; Table S4: Association between atopic diseases and anemia with complete dataset; Table S5: Association between the severity of allergic diseases and anemia; Table S6: Subgroup analysis in a cohort that excluded children having been diagnosed with anemia by a doctor at 1 year of age according to caregiver’s report; Table S7: Comparison of variables between data used for analysis and data of those lost to follow-up at 3 years of age; Table S8: Logistic regression model with whether lost follow-up as dependent variable.
