# Supplementary material for: Allergic Disorders and Risk of Anemia in Japanese Children: Findings from the Japan Environment and Children’s Study
Source: Nutrients. 2022 Oct 17;14(20):4335. doi: 10.3390/nu14204335 (PMC9607270; doi:10.3390/nu14204335)
Supplement: Supplementary file 1 [file nutrients-14-04335-s001.zip › nutrients-1939684-supplementary.pdf]

Table S1. Definitions of allergic diseases

**Asthma**	A positive answer to the question “Has your child ever been diagnosed by a physician as having asthma in the past 6 months?” asked at children aged 1.5 years or 2 years.
**Atopic dermatitis**	A positive answer to both the following question:<br>1) “Has your child had this itchy rash at any time in the past 12 months?”<br>2) “Has this itchy rash at any time affected any of the following places: the folds of the elbows, behind the knees, in front of the ankles, under the buttocks, or around the neck, ears, or eyes?”
**Allergic rhinitis**	A positive answer for both of the following questions: “In the past 12 months, has your child had a problem with sneezing, or a runny or blocked nose when he/she did not have a cold or the flu?”
**Allergic rhinoconjunctivitis**	Allergic rhinitis combined with itchy, tearful eyes.
**Food allergy**	A positive answer to the question “Has your child ever been diagnosed by a physician as having food allergy in the past 6 months?” asked at children aged 1.5 years or 2 years.

Table S2. Baseline characteristics of the study cohort

		Total		Anemia at 3 years (-)			
				No		Yes	
**Variables**		**N**	**%**	**N**	**%**	**N**	**%**
Smoking exposure	No	52440	67.1	52251	67.1	189	70.8
	Yes	25712	32.9	25634	32.9	78	29.2
	Missing	2791		2776		15	
Maternal allergic history	No	33610	41.8	33506	41.8	104	36.9
	Yes	46887	58.2	46709	58.2	178	63.1
	Missing	446		446		0	
Maternal education level	High	52857	66	52656	66	201	72.8
	Low	27174	34	27099	34	75	27.2
	Missing	912		906		6	
Paternal education level	High	46108	57.9	45946	57.9	162	59.1
	Low	33513	42.1	33401	42.1	112	40.9
	Missing	1322		1314		8	
Family incomes	High	45968	61.1	45804	61.1	164	63.8
	Low	29283	38.9	29190	38.9	93	36.2
	Missing	5692		5667		25	
Pet keeping	No	62227	77.7	62001	77.7	226	81.9
	Yes	17853	22.3	17803	22.3	50	18.1
	Missing	863		857		6	
Birth weight	>=2500g	74349	92.1	74114	92.1	235	84.2
	<2500g	6388	7.9	6344	7.9	44	15.8
	Missing	206		203		3	
Sex	Girls	39460	48.8	39378	48.8	82	29.1
	Boys	41483	51.2	41283	51.2	200	70.9
Premature birth	No	77109	95.4	76859	95.5	250	89.3
	Yes	3676	4.6	3646	4.5	30	10.7
	Missing	158		156		2	

Breastfeeding	No	50298	63.1	50165	63.2	133	47.8
	Yes	29356	36.9	29211	36.8	145	52.2
	Missing	1289		1285		4	
Sleeping time	>=11.5h	62742	79.6	62532	79.6	210	76.6
	<11.5h	16115	20.4	16051	20.4	64	23.4
	Missing	2086		2078		8	
Kindergarten	No	39586	50.7	39474	50.8	112	40.7
	Yes	38423	49.3	38260	49.2	163	59.3
	Missing	2934		2927		7	
Parity	No	32893	41.7	32788	41.7	105	38.7
	Yes	46073	58.3	45907	58.3	166	61.3
	Missing	1977		1966		11	
Allergic diseases at 2 years							
Asthma	No	74005	92.5	73765	92.5	240	85.7
	Yes	5988	7.5	5948	7.5	40	14.3
	Missing	950		948		2	
Atopic dermatitis	No	67975	87	67771	87	204	73.9
	Yes	10199	13	10127	13	72	26.1
	Missing	2769		2763		6	
Allergic rhinitis	No	55748	71.8	55572	71.8	176	64.7
	Yes	21898	28.2	21802	28.2	96	35.3
	Missing	3297		3287		10	
Allergic rhinoconjunctivitis	No	75268	97.1	75020	97.1	248	91.5
	Yes	2229	2.9	2206	2.9	23	8.5
	Missing	3446		3435		11	
Food allergy	No	71111	88.9	70891	88.9	220	78.6
	Yes	8882	11.1	8822	11.1	60	21.4
	Missing	950		948		2	
Any allergy	No	45026	56.3	44914	56.3	112	40
	Yes	34967	43.7	34799	43.7	168	60
	Missing	950		948		2	
Anemia at 3 years	No	80661	99.7		100		
	Yes	282	0.3				

Table S3. Standardized mean difference after weighing in the IPTW process

	Asthma	Atopic dermatitis	Allergic rhinitis	Allergic rhinoconjunctivitis	Food allergy	Any allergy
Smoking exposure	0.004	0.001	0.003	0.006	0.007	0.001
Maternal allergic history	0.002	0.002	0.001	0.009	0.006	<0.001
Maternal education level	0.02	0.004	0.001	0.009	0.011	0.001
Paternal education level	<0.001	0.003	0.001	0.010	0.004	0.001
Family incomes	0.012	<0.001	0.003	0.016	<0.001	0.001
Pet keeping	0.001	0.001	0.001	0.003	0.003	<0.001
Birth weight	0.008	0.001	0.001	0.011	0.01	<0.001
Sex	0.025	0.001	0.002	0.019	0.006	0.001
Premature birth	0.003	0.005	0.001	0.002	0.007	0.001
Breastfeeding	0.025	<0.001	0.002	0.008	0.003	0.001
Sleeping time	0.015	0.001	0.002	0.014	0.003	<0.001
Kindergarten	0.042	0.002	<0.001	0.016	0.007	<0.001
Parity	0.054	0.001	<0.001	0.012	0.006	<0.001

Table S4. Association between atopic diseases and anemia with complete dataset

		Model 1#			Model 2$		
			95% CI			95% CI	
**Outcome events**		**ORs**	**Lower**	**Upper**	**ORs**	**Lower**	**Upper**
Asthma	Yes vs. No	1.69	1.15	2.47	1.73	1.17	2.56
Atopic dermatitis	Yes vs. No	2.38	1.79	3.17	2.42	1.81	3.24
Allergic rhinitis	Yes vs. No	1.38	1.05	1.81	1.38	1.05	1.81
Allergic rhinoconjunctivitis	Yes vs. No	2.96	1.84	4.75	3.05	1.90	4.90
Food allergy	Yes vs. No	1.98	1.45	2.70	1.88	1.37	2.59
Any allergy	Yes vs. No	1.88	1.44	2.45	1.83	1.40	2.39
Atopic diseases, No.	1 vs. 0	1.45	1.07	1.96	1.38	1.01	1.88
	>1 vs. 0	2.95	2.14	4.07	2.96	2.14	4.09

Abbreviations: OR, odds ratio; CI: confidence interval; BMI, body mass index.

#The model adjusted smoking exposure, parental education levels, family income, birth weight, sex, premature birth, breastfeeding, sleeping time, and kindergarten.

$ Except those adjusted in model 1, the models further adjusted the z scores of BMI at 2 years of age.

Table S5. Association between the severity of allergic diseases and anemia

		Model 1#			Model 2$		
			95% CI			95% CI	
		ORs	Lower	Upper	ORs	Lower	Upper
Atopic dermatitis							
Sleeping disturbance	Yes vs. No	1.95	1.22	3.10	1.94	1.22	3.10
Rhinitis							
Having interference in daily life	Severe vs. non or mild	2.52	1.26	5.02	2.50	1.25	5.00

Abbreviations: OR, odds ratio; CI: confidence interval; BMI, body mass index.

#The model adjusted sex, premature birth, breastfeeding, and sleeping time.

$Except those adjusted in model 1, the models further adjusted the z scores of BMI at 2 years of age.

Table S6. Subgroup analysis in a cohort that excluded children having been diagnosed with anemia by a doctor at 1 year of age according to caregiver's report

		Model 1 #			Model 2 $		
			95% CI			95% CI	
**Outcome events**		**ORs**	**Lower**	**Upper**	**ORs**	**Lower**	**Upper**
Asthma	Yes vs. No	1.78	1.18	2.66	1.83	1.22	2.75
Atopic dermatitis	Yes vs. No	2.19	1.59	3.00	2.17	1.58	2.98
Allergic rhinitis	Yes vs. No	1.51	1.13	2.01	1.52	1.14	2.02
Allergic rhinoconjunctivitis	Yes vs. No	3.05	1.85	5.03	3.04	1.84	5.01
Food allergy	Yes vs. No	1.67	1.18	2.38	1.65	1.16	2.35
Any allergy	Yes vs. No	1.80	1.36	2.38	1.81	1.37	2.39
Atopic diseases, No.	1 vs. 0	1.38	1.05	1.82	1.39	1.05	1.83
	>1 vs. 0	2.84	2.12	3.82	2.86	2.13	3.84

Abbreviations: OR, odds ratio; CI, confidence interval; BMI, body mass index.

The models adjusted smoking exposure, parental education levels, family income, birth weight, sex, premature birth, breastfeeding, sleeping time, and kindergarten.

$ Except those adjusted in model 1, the models further adjusted the z scores of BMI at 2 years of age.

Table S7. Comparison of variables between data used for analysis and data of those lost to follow-up at 3 years of age

		Lost to follow-up		Data used for analysis		
**Variables**		**N**	**%**	**N**	**%**	**p**
Smoking exposure	Yes	3449	44.9	25712	32.9	<0.001
Maternal allergic history	Yes	9331	56.1	46887	58.2	<0.001
Maternal education level	Low	7748	48.1	27174	34.0	<0.001
Paternal education level	Low	8662	54.5	33513	42.1	<0.001
Family incomes	<400	6863	47.2	29283	38.9	<0.001
Pet keeping	Yes	4331	26.9	17853	22.3	<0.001
Birth weight	<2500g	1573	9.1	6388	7.9	<0.001
Sex	Boys	8951	51.3	41483	51.2	0.92
Premature birth	Yes	940	5.4	3676	4.6	<0.001
Breastfeeding	Yes	3836	32.2	29356	36.9	<0.001
Sleeping time	<11.5h	1811	23.2	16115	20.4	<0.001
Kindergarten	Yes	3259	55.7	38423	49.3	<0.001
Parity	≥1	11287	66.2	46073	58.3	<0.001
Allergic diseases at 2 years						
Asthma	Yes	712	8.3	5988	7.5	<0.007
Atopic dermatitis	Yes	794	13.5	10199	13.0	0.275
Allergic rhinitis	Yes	1763	30.3	21898	28.2	<0.001
Allergic rhinoconjunctivitis	Yes	191	3.3	2229	2.9	0.069
Food allergy	Yes	847	9.9	8882	11.1	<0.001
Any allergy	Yes	3148	36.7	34967	43.7	<0.001

Table S8. Logistic regression model with whether lost follow-up as dependent variable

Independent variables	Coefficients	p
Smoking exposure	0.038	<0.001
Maternal allergic history	0.036	0.416
Maternal education level	0.04	<0.001
Paternal education level	0.039	0.001
Family incomes	0.037	0.012
Pet keeping	0.042	0.008
Birth weight	0.075	0.752
Sex	0.036	0.42
Premature birth	0.097	0.613
Breastfeeding	0.038	0.013
Sleeping time	0.043	0.109
Kindergarten	0.037	<0.001
Parity	0.037	<0.001
Allergic diseases at 2 years	0.036	0.526
